# Association between mental health disorders and asthma exacerbations in adults: a retrospective cohort study in UK primary care

**DOI:** 10.1136/bmjresp-2025-003244

**Published:** 2025-11-17

**Authors:** Tris Pickard-Michels, Nicola J Adderley, Prasad Nagakumar, Nikita Simms-Williams, Alice Sitch, Rasiah Thayakaran, Dhanusha Punyadasa, Krishnarajah Nirantharakumar, Shamil Haroon

**Affiliations:** 1 University of Birmingham, Birmingham, UK; 2 Institute of Applied Health Research, University of Birmingham, Birmingham, UK; 3 Respiratory Medicine, Birmingham Women’s and Children’s Hospitals NHS Foundation Trust, Birmingham, UK; 4 Institute of Inflammation and Ageing University, University of Birmingham, Birmingham, UK; 5 University of Birmingham Unit of Public Health Epidemiology and Biostatistics, Birmingham, UK; 6 University of Birmingham Institute of Applied Health Research, Birmingham, UK; 7 Public Health, University of Birmingham, Birmingham, UK; 8 Health and Population Sciences, University of Birmingham, Birmingham, UK

**Keywords:** Asthma, Asthma Epidemiology, Asthma in primary care

## Abstract

**Aim:**

To evaluate the association between the presence or absence of comorbid mental health disorders and the risk of asthma exacerbations in adults with prevalent asthma.

**Methods:**

This was a cohort study of adults in England with prevalent asthma and mental health disorders (depression, anxiety, bipolar disorder and schizophrenia) between 2017 and 2019 using primary care electronic healthcare records.

Adult asthma patients with mental health disorders (exposed) were matched by age, sex, ethnicity and general practice to asthma patients without a mental health disorder (unexposed) in a 1:1 ratio. The primary outcome was an exacerbation of asthma documented in primary care records. Poisson regression was used to estimate adjusted incidence rate ratios (IRR).

**Results:**

873 482 adults with asthma were followed up for a total of 1 580 157 years.

Mean age was 49 years; 66% were female, and 78% were white. Adults with asthma and any mental health disorder had an asthma exacerbation incidence rate of 56 per 1000 person years compared with 34 per 1000 person years for those without a mental health disorder.

Adults with asthma and any mental health disorder had an adjusted IRR of 1.46 (95% CI 1.44 to 1.48) compared with matched controls. The highest IRR was in those with depression (IRR 1.34, 95% CI 1.32 to 1.37), followed by those with anxiety (IRR 1.20, 95% CI 1.18 to 1.22). There were no significant differences in patients with bipolar disorder or schizophrenia compared with matched controls (IRR 1.00, 95%CI 0.93 to 1.07; and 1.03, 95% CI 0.95 to 1.11, respectively).

**Conclusion:**

This study shows a significant increased risk of asthma exacerbations in asthma patients with depression or anxiety compared with those with asthma without comorbid mental health disorders.

**WHAT IS ALREADY KNOWN ON THIS TOPIC**

- There is a recognised association between asthma exacerbations (AEs) and mental health disorders; however, this has not yet been quantified in the UK primary care population.

**WHAT THIS STUDY ADDS**

- Having comorbid depression or anxiety is associated with a significantly increased risk of AEs among adults with asthma in the UK.

**How this study might affect research, practice or policy**

- The increase in AE incidence rate among asthma patients with comorbid mental health disorders, as shown in this study, suggests that initiatives to reduce AE incidence rates may be more effective if they focus on this group.

## Introduction

 Asthma is one of most common causes of morbidity in England,^1^ with a national prevalence of 6%.^1^,^3^ There is increasing recognition that comorbid conditions have a negative effect on asthma outcomes.^4^,^8^ Mental health disorders are common and represent a significant proportion of asthma-related comorbidities. The prevalence of depression and anxiety in the UK general population is 16% and 14%, respectively,^9^ whereas those with asthma have a 60% increased risk of depression and a 50% increased risk of anxiety as reported in the 2017 World Mental Health Survey.^10^ Despite this association, there is limited and conflicting literature on asthma-related outcomes in this group.^11^

Although the burden of asthma management mainly falls within primary care (74% of the total economic cost), there are no large, UK-based, primary care studies on asthma outcomes associated with comorbid mental health disorders. It is recognised that policies need to address this disproportionate service load as part of managing the problem of acute asthma exacerbations (AE).^12^

Of the few primary studies identified, more were cross-sectional^13^,^18^ than longitudinal^19^,^23^ in design. Furthermore, many studies were small (n=85–400 participants) and set in secondary care.^13^,^2023^ Several small studies have demonstrated conflicting results.^15 17 20^ Of these, a Canadian retrospective cohort study of 406 asthma patients^26^ used subjective and objective asthma outcome measures. The Asthma Control Questionnaire and Asthma Quality of Life Questionnaire showed that the asthma patients exposed to comorbid mental illness had significantly worse outcomes compared with those without, whereas there was no significant difference in forced expiratory volume in 1 second (FeV1) and forced vital capacity (FVC) between these groups.

Similarly, an American prospective study of 568 asthma patients showed conflicting asthma-related outcome measures for those exposed to depression. This study used self-reported measures of exposure to depression and additionally surrogate asthma outcome measures of service utilisation and drug prescription counts. The results showed an association between depressive symptoms and an increase in asthma-related emergency department visits, but no association with the frequency of oral corticosteroid prescriptions.^19^

Two large cohort studies from Sweden and the USA (25 000–34 000 participants) used electronic healthcare record^21 23^ linking primary and secondary care records to assess surrogate measures of AE. These studies by Lisspers and Sumino *et al* showed an increase in AE in those exposed to asthma with comorbid anxiety and depression.

Finally, all studies using surrogate outcomes of healthcare utilisation in secondary care are unlikely to be generalisable to primary care.^19 24 26^

## Method

### Ethics

The protocol was submitted to and approved by the Clinical Practice Research Datalink (CPRD) Independent Scientific Advisory Committee – protocol number *22_001*8*37.* The Health Research Authority ethics approval was provided to use CPRD data.^27 28^

### Patient and public involvement

Patients and public were not involved in the design or dissemination of this study which used anonymised CPRD data.

### Study design

A retrospective matched cohort study was conducted using routinely collected primary care data from the CPRD Aurum database from 1 January 2017 to 1 January 2019.^27 28^

Eligible patients were adults aged 18 years and older, registered with a GP (General Practice) for more than 1 year prior to the index date (1 January 2017) and with a clinically coded diagnosis for asthma prior to the study start date. The exposures of interest were comorbid mental health disorders diagnosed 1 year prior to the study. These included mental health disorders with the highest overall morbidity: depression, anxiety, bipolar disorder and schizophrenia. Adults with asthma and mental health conditions were directly matched to unexposed controls (those without a recorded mental health condition) in a 1:1 ratio by age, sex, ethnicity and GP practice.

Adults from the control group who developed mental health disorders during the follow-up period were excluded from the study. The study follow-up period was from 1 January 2017 to 1 January 2019.

### Outcomes

The outcome measure was incident asthma exacerbations recorded in primary care (see online supplemental appendix 1 for the relevant SNOMED-CT codes).

### Validation of outcome measure

AEs are heterogenous in nature which requires clinical evaluation to diagnose accurately.^29^ This study uses a SNOMED-CT coded diagnosis of AE, recorded by a health professional.

### Study power and sample size

The minimally important difference for asthma exacerbations in this study was set at 10% relative difference in asthma exacerbations between exposed and unexposed groups. A two-sided significance level of 0.05 was used, with a 1:1 ratio of exposed to unexposed. Baseline asthma exacerbation percentage prevalence was taken as 0.059.^12^ The minimum total sample size calculated was 70 162 to achieve 90% power. The dataset included 873 482 participants, which was sufficient to achieve this power.

### Development and validation of study code lists

Code lists were developed a priori and validated through a consensus between specialists in primary care and public health practitioners. These included Quality and Outcomes Framework indicator codes^2^, where a systematic process, using an in-house software tool called Code Builder, generated the clinical code lists for depression, anxiety, bipolar disease, schizophrenia and asthma exacerbation (online supplemental appendix 1).

### Data cleaning and handling

The data were inspected to assess the number of missing or incorrect entries. Missing-category variables were created (and designated the highest number for each group) to include missing or erroneous data entries to avoid losing data which could have biased the results. Categorical variables were created for missing or erroneous/implausible data entries. The absence of a diagnostic code was assumed to indicate the absence of the condition.

As defined in previous asthma research, age was categorised into the following groups: 18–54 and 55 years, to allow for potential confounding in the older age group from a chronic obstructive pulmonary disease (COPD) diagnosis.^30^ Body mass index (BMI) data which had implausible outlier entries (BMI <10 kg/m^2^, BMI >200 kg/m^2^) were replaced as missing data. BMI was categorised into underweight (<18.5 kg/m^2^) normal weight (18.5–25 kg/m^2^), overweight (25–30 kg/m^2^) and obese (>30 kg/m^2^). Ethnicity categories were White, Asian, Black, Mixed and Other. Smoking status categories were never-smoker, current smoker and ex-smoker.

### Statistical analysis

The primary outcome (AE incidence rate) was calculated and further stratified by mental health disorder subgroups.

A Poisson regression model was used to assess the association between the primary outcome and the presence of a comorbid mental disorder.^1^ AEs were considered independent of each other. A univariable Poisson model was fitted, producing AE incidence rate ratios by exposure to mental health disorders. Multivariable Poisson regression models were fitted to adjust for potential confounders.

The covariates included in the model were: demographic variables (age, sex and ethnicity), behavioural characteristics (BMI and smoking status (10,69)), medical comorbidities (allergies, allergic rhinitis, atopic dermatitis, chronic rhinosinusitis, gastro-oesophageal reflux disease (GORD), obstructive sleep apnoea, bronchiectasis (non-cystic fibrosis), interstitial lung disease, COPD, cardiovascular disease, diabetes mellitus) and prescriptions of beta-blockers and non-steroidal anti-inflammatory drugs (NSAIDs) as these have a known association with AEs.^31 32^

Model A was adjusted for demographic and behavioural characteristics, and model A extended was additionally adjusted for comorbidities. Exploratory regression models were fitted (Model B, Model B extended) to investigate the additional effect of mental disorder subgroups on the incidence rate ratio.

Additionally, the interaction between depression and anxiety dual diagnoses was investigated, adjusting for demographic and behavioural characteristics with and without comorbidities.

Analysis was performed using STATA/SE V.16.1.

## Results

The study population included 873 482 participants with a mean age of 49 years. Total person years of follow-up was 1 580 157 person years. Baseline demographic and clinical characteristics were described by exposure status (table 1).

**Table 1:** Baseline characteristics of asthma patients by exposure to mental health disorders

Characteristics	No mental health disorder	Mental health disorder	Total
Number, n (%)	436 741 (50.0)	436 741 (50.0)	873 482 (100.0)
Age, (years) mean (SD)	49.14 (19.2)	48.89 (17.2)	49.02 (18.2)
Sex, n (%)			
Male	146 811 (33.6)	147 128 (33.7)	293 939 (33.7)
Female	289 930 (66.4)	289 613 (66.3)	579 543 (66.4)
Ethnicity, n (%)			
White	342 065 (78.3)	344 872 (79.0)	686 937 (78.6)
Mixed	4231 (1.0)	4450 (1.0)	8681 (1.0)
Asian	16 059 (3.7)	15 683 (3.6)	31 742 (3.6)
Black	8994 (2.1)	8466 (1.9)	17 460 (2.0)
Other	2187 (0.5)	2046 (0.5)	4233 (0.5)
Missing	63 205 (14.5)	61 224 (14.0)	124 129 (14.3)
BMI (kg/m^2^) n (%)			
<18.5	11 474 (2.6)	11 386 (2.6)	22 860 (2.6)
18.5–25	148 486 (34.0)	129 939 (29.8)	278 425 (31.9)
25–30	126 624 (29.0)	123 917 (28.4)	250 541 (28.7)
>30	111 929 (25.6)	143 395 (32.8)	255 324 (29.2)
Missing	38 228 (8.8)	28 104 (6.4)	66 332 (7.6)
Smoking, n (%)			
Never smoker	129 701 (32.6)	90 709 (22.2)	220 410 (27.3)
Ex-smoker	172 301 (43.2)	172 021 (42.1)	344 322 (42.7)
Smoker	95 296 (23.9)	145 325 (35.6)	240 621 (29.8)
Missing	5099 (1.2)	1930 (0.4)	7029 (0.8)
Comorbidities, n (%)			
Allergies	111 682 (25.6)	125 995 (28.9)	237 677 (27.2)
Allergic rhinitis	129 562 (29.7)	142 495 (32.6)	272 057 (31.2)
Atopic eczema	110 084 (25.2)	121 183 (27.8)	231 267 (26.5)
Bronchiectasis	7276 (1.7)	7424 (1.7)	14 700 (1.7)
Chronic sinusitis	9992 (2.3)	15 368 (3.5)	25 360 (2.9)
COPD	39 348 (8.8)	50 504 (11.6)	88 852 (10.2)
Diabetes type 1	2274 (0.5)	3293 (0.8)	5567 (0.6)
Diabetes type 2	29 868 (6.8)	40, 230 (9.2)	70 098 (8.0)
GORD	70 371 (16.1)	42 907 (9.8)	113 278 (13.0)
Heart failure	8314 (1.9)	9192 (2.1)	17 506 (2.0)
Myocardial infarction	6938 (1.6)	8864 (2.0)	15 802 (1.8)
Ischaemic heart disease	21 064 (4.8)	27 642 (6.3)	48 706 (5.6)
Total stroke	9546 (2.2)	13 239 (3.0)	22 785 (2.6)
interstitial lung disease	1204 (0.3)	1436 (0.3)	2640 (0.3)
Obstructive sleep apnoea	4206 (1.0)	10 088 (2.3)	14 294 (1.6)
Peripheral vascular disease	2590 (0.6)	3727 (0.9)	6317 (0.7)
Prescriptions, n (%)			
Beta-blockers	21 105 (4.8)	30 422 (7.0)	51 527 (5.9)
NSAID	146 136 (33.5)	196 828 (45.1)	342 964 (39.3)

BMI, body mass index; COPD, chronic obstructive airways disease; GORD, gastro-oesophageal reflux disease; NSAID, Non-steroidal anti-inflammatory drug.

Depression and anxiety were the most prevalent types of mental health disorders with 346 474 (80% of the exposed population) and 270 664 (62%), respectively. Bipolar and schizophrenia were each less than 2% of the exposed population. Many patients in the exposed group had more than one mental health diagnosis, in particular anxiety with depression, 184 642 (42.3%).

A total of 70 316 asthma exacerbations were observed with an IR of 40 per 1000 person years for the total study population (table 2).

**Table 2:** Asthma exacerbation (AE) incidence rate stratified by mental health disorder subgroups

Diagnosis	AE	Person years	AE crude incidence rate per 1000 person years (95% CI)
Without mental health disorder	26 597	793 865	34 (33 to 34)
With mental health disorder	43 719	786 292	56 (55 to 56)
Depression	36 669	623 488	59 (58 to 59)
Anxiety	27 814	487 578	57 (56 to 58)
Bipolar disorder	860	15 089	57 (53 to 61)
Schizophrenia	718	14 406	50 (46 to 54)

The unadjusted Poisson regression analysis showed a 66% increase in the AE incidence rate for all mental health disorders compared with matched controls (incidence rate ratio (IRR) 1.66,95% CI 1.63 to 1.69) (table 3).

**Table 3:** Unadjusted Poisson regression analysis. Incidence rate ratios (IRR) for acute asthma exacerbations in the exposed group compared with the unexposed group

Diagnosis	IRR	95% CI	P value
All mental health disorders	1.66	(1.63 to 1.69)	<0.001
Depression	1.67	(1.65 to 1.70)	<0.001
Anxiety	1.47	(1.44 to 1.49)	<0.001
Bipolar	1.28	(1.20 to 1.37)	<0.001
Schizophrenia	1.12	(1.04 to 1.21)	0.003

After adjusting for age, sex, ethnicity, BMI and smoking (model A), those with any mental disorder had a 55% increase in AE incidence rate compared with matched controls (IRR 1.55, 95% CI 1.52 to 1.57) (table 4). With additional adjustment for comorbidities (model A extended), those with any mental health disorder had adjusted IRR of 1.46 (95% CI 1.44 to 1.48) compared with matched controls. Adjusted exploratory analyses (model B) indicated an adjusted IRR of 1.39 (95% CI 1.37 to 1.42) for depression and 1.24 (95% CI 1.22 to 1.26) for anxiety compared with those without these disorders. With additional adjustment for comorbidities, results were similar.

**Table 4:** Adjusted Poisson analysis

Diagnoses	Model A	A extended	Model B	B extended
	IRR (95% CI)	IRR (95% CI)	IRR (95% CI)	IRR (95% CI)
All mental health disorders	1.55 (1.52, 1.57)	1.46 (1.44.1.48)		
Depression			1.39 (1.37, 1.42)	1.34 (1.32, 1.37)
Anxiety			1.24 (1.22, 1.26)	1.20 (1.18, 1.22)
Bipolar disorder			1.00 (0.93, 1.07)	1.00 (0.93, 1.07)
Schizophrenia			1.01 (0.93, 1.09)	1.03 (0.95, 1.11)
Age (years)*				
55+	1.23 (1.11, 1.14)	1.11 (1.09, 1.12)	1.23 (1.11, 1.15)	1.11 (1.09, 1.13)
Sex†				
Female	1.80 (1.77, 1.83)	1.75 (1.72, 1.78)	1.77 (1.74, 1.81)	1.73 (1.70, 1.76)
Ethnicity‡				
Mixed race	0.93 (0.86, 1.01)	0.90 (0.83, 0.97)	0.94 (0.87, 1.02)	0.91 (0.84, 0.98)
Asian	1.58 (1.53, 1.64)	1.44 (1.39, 1.49)	1.60 (1.55, 1.66)	1.46 (1.41, 1.51)
Black	0.97 (0.93, 1.02)	0.91 (0.87, 0.96)	0.99 (0.94, 1.04)	0.93 (0.88, 0.98)
Others	1.06 (0.96, 1.18)	1.03 (0.92,1.14)	1.07 (0.96, 1.18)	1.03 (0.92, 1.14)
Missing	0.75 (0.73, 0.77)	0.75 (0.73, 0.77)	0.76 (0.74, 0.78)	0.76 (0.74, 0.77)
BMI (kgm^−2^)§				
18.5–25	1.39 (1.30, 1.49)	1.37 (1.28, 1.46)	1.39 (1.30, 1.49)	1.36 (1.27, 1.46)
25–30	1.93 (1.81, 2.07)	1.86 (1.74, 1.99)	1.93 (1.80, 2.06)	1.86 (1.73, 1.99)
30+	2.81 (2.63, 3.01)	2.66 (2.48, 2.84)	2.79 (2.61, 2.99)	2.64 (2.47, 2.83)
Missing	1.10 (1.01, 1.19)	1.12 (1.03, 1.21)	1.10 (1.01, 1.19)	1.11 (1.03, 1.21)
Smoking status¶				
Ex-smoker	1.18 (1.15, 1.20)	1.16 (1.13, 1.18)	1.17 (1.15, 1.20)	1.15 (1.13, 1.18)
Current smoker	1.20 (1.18, 1.23)	1.22 (1.19, 1.24)	1.19 (1.17, 1.22)	1.21 (1.19, 1.24)
Missing	0.26 (0.20, 0.34)	0.29 (0.22, 0.37)	0.26 (0.20, 0.34)	0.28 (0.22, 0.37)
Comorbidities				
Allergies		1.22 (1.20, 1.25)		1.22 (1.20, 1.25)
Allergic rhinitis		1.17 (1.14, 1.19)		1.17 (1.14, 1.19)
Atopic eczema		1.16 (1.14, 1.17)		1.15 (1.14, 1.17)
Bronchiectasis		1.32 (1.26, 1.39)		1.32 (1.26, 1.39)
Chronic sinusitis		1.34 (1.30, 1.39)		1.34 (1.29, 1.38)
COPD		0.93 (0,91, 0.96)		0.93 (0.91, 0.96)
Type 2 diabetes		1.06 (1.03, 1.08)		1.06 (1.03, 1.08)
Type 1 diabetes		0.87 (0.79, 0.95)		0.86 (0.79, 0.95)
GORD		1.30 (1.27, 1.32)		1.29 (1.27, 1.32)
Heart failure		1.01 (0.96, 1.06)		1.01 (0.96, 1.07)
Myocardial infarction		0.91 (0.85, 0.97)		0.91 (0.85, 0.97)
Ischaemic heart disease		0.99 (0.95, 1.02)		0.99 (0.95, 1.02)
Interstitial lung disease		1.00 (0.88, 1.14)		1.00 (0.88, 1.14)
Obstructive sleep apnoea		1.28 (1.22, 1.34)		1.27 (1.21, 1.33)
Peripheral vascular disease		0.68 (0.62,0.76)		0.68 (0.62, 0.76)
Betablockers		0.76 (0.73, 0.78)		0.76 (0.73, 0.78)
NSAIDs		1.16 (1.14, 1.18)		1.16 (1.14, 1.18)

Incidence rate ratios (IRR) for acute asthma exacerbations in asthma patients with comorbid mental health conditions compared with those without.

Model A: all mental health disorders adjusted for age, sex, ethnicity, BMI and smoking status.

Model B: mental health disorder subgroups adjusted for age, sex, ethnicity, BMI and smoking status.

Model A and B extended additionally adjusted for comorbidities.

* 18–54.

† Male.

‡ White.

§ <18.5.

¶ Non-smoker.

BMI, body mass index; COPD, chronic obstructive airways disease; GORD, gastro-oesophageal reflux disease; NSAID, non-steroidal anti-inflammatory drugs.

There was a significant relative increase in incidence rate of AE for Asian groups, 1.44 (CI 1.39, 1.49) compared with White ethnicity. In contrast, there was a significant relative reduction in incidence rate of AE for Black, 0.91 (CI 0.87 to 0.96), and Mixed, 0.90 (CI 0.83 to 0.97), compared with White ethnicity.

Dispersion and deviance were assessed in the Poisson model using the Pearson and deviance χ2 tests, which were 0.0000 and 1.0000, respectively, which confirms that the statistical model was an appropriate fit for the data.

The interaction analysis, for the dual diagnosis of depression and anxiety, adjusted for behavioural characteristics and comorbidities, showed a 40% (CI 1.38 to 1.43) increase in AE incidence rate for adults with asthma and comorbid depression compared with those without comorbid depression.

In addition, adults with asthma and comorbid anxiety showed a 30% (CI 1.26 to 1.33) increase in AE incidence rate compared with asthma patients without comorbid anxiety. The interaction analysis showed that in participants with depression and anxiety, the combined effect (interaction coefficient 0.88 (95% CI 0.85 to 0.91)) was slightly less than the sum of the individual effects (table 5).

**Table 5:** Adjusted analysis with interaction term for depression and anxiety

Mental disorder exposure	IRR	95% (CI)
Depression alone	1.40	1.38, 1.43
Anxiety alone	1.30	1.26, 1.33
Depression and anxiety interaction	0.88	0.85, 0.91

IRR, incidence rate ratios.

## Discussion

### Key results

This study presents asthma exacerbation incidence rates for 873 482 adults in UK primary care with prevalent asthma among those with a comorbid mental health condition and those without. Asthma patients with a mental health disorder had a 66% higher incidence of AE compared with those without a mental health disorder before adjustment for potential confounders. In addition, all subgroups of mental health disorder had higher unadjusted incidence of AE compared with matched controls. Asthma with depression had the highest incidence rate of AE. After adjusting for age, sex, ethnicity, BMI, smoking status, comorbidities and prescriptions, the incidence rate of AE for those with asthma and all mental health disorders remained significantly increased at 46%. After adjustment, the AE incidence rate remained significantly increased for the depression and anxiety mental disorder subgroups; however, for bipolar and schizophrenia, the increased incidence of AE disappeared.

### Relationship to other studies

Heterogeneous outcome measures of AE hindered comparison of this study to previous research. The AE outcome measures in previous studies ranged from subjective self-reported asthma symptoms using asthma control questionnaires to objective measures of lung function tests and surrogate measures of oral corticosteroid prescriptions or healthcare utilisation measures. In addition, these were small cross-sectional studies.^13^,^1517^

The only two large, published cohort studies^21 23^ used composite AE outcome measures of healthcare utilisation. Our study used professionally diagnosed and coded AE outcomes in primary care. While outcome measures from the above studies are not directly comparable, our study had a relative risk of AE in adults with a comorbid mental health disorder which was higher than that found in either of the previous cohort studies.

As seen in previous studies, overlap of the mental health disorder subgroups was noted, with participants frequently having more than one mental health disorder diagnosis.^15 18^ In this study, 346 474 of the participants had a depression diagnosis, and of these, 184 642 had anxiety as a second diagnosis.

We did not see a significant increase in adjusted AE incidence rate for asthma patients with comorbid schizophrenia or bipolar disorder. Mental health disorders are documented less consistently than asthma, particularly schizophrenia and bipolar disorders, due to concerns over stigmatisation and uncertainty over evolving diagnosis.^33^ It may be that this group of patients is not attending primary care and therefore the AEs are not being captured. Additionally, the effect of a second diagnosis complicates the picture, and separating out the individual diagnoses loses power.

In considering the interaction of comorbid depression with anxiety in asthma patients the findings of our study are in line with a previous cross-sectional study^18^ showing that there is an additive effect on relative risk in patients with both diagnoses.

Previous research has shown that ethnic minority groups have worse asthma outcomes due to issues surrounding unequal access to care, cultural factors including language barriers^34^ and socioeconomic status, which has been shown to be lower in ethnic minorities.^35^ We found a significant relative increase in the incidence rate of AE for Asian groups (44%) compared with White ethnicity, which is consistent with previous research.^34^,^36^ In contrast to previous research,^36^ Black and Mixed populations in our study showed a significant relative reduction in incidence rate of AE compared with White ethnicity.^34 35^ Inconsistent or missing documentation of ethnicity may have underrepresented this group of asthma patients.^34^

Evidence suggests an association between schizophrenia and asthma via a common autoimmune process which could make this comorbid group susceptible to worse asthma outcomes.^37^ This study did not show a relative increase in incidence rate for asthma with comorbid schizophrenia and bipolar disorder; however, these outcomes were complicated by a high level of multiple mental disorder diagnoses, which merits further investigation.

The findings of this study can be supported by research in psychoimmunology which suggests several biological mechanisms potentially linking mental health with airway inflammation.^38^ In addition, adults with mental health disorders may not access routine healthcare and may have poor adherence to treatment regimens.^39^

### Strengths and limitations

This is the first UK-based study of asthma with mental health disorders, using high quality primary care data, representative of the population of England and therefore generalisable to UK primary care management of these patients.

A key strength of the study design is the inclusion criteria of pre-existing asthma and mental health diagnoses which reduces reverse causality. However, asthma patients who have frequent exacerbations may consequently develop depression or anxiety, and therefore bidirectional relationships cannot be fully excluded.

The retrospective design affected the availability of pre-collected data for relevant potential confounders ethnicity and BMI (14% and 8% missing, respectively). The assumption was that this data was missing at random, which, if violated, could have introduced bias, affecting internal validity. Deprivation is a potential confounder for AEs; however, without linked socioeconomic data, adjusting for this in the analysis was not possible which may have biased the results by overestimating the effect of mental health disorder exposure on AEs.

AEs were assumed to be independent of the timings of diagnosis of asthma and mental health condition, prior to the study start date. Exposure to mental health disorders was assumed to be accurately documented for the follow-up period; however, any mental health disorders which may have resolved in the exposed group or been undiagnosed in the control group would introduce misclassification bias, either of which would reduce the size of the effect estimate.

Asthma severity was not adjusted for and may have introduced bias with the potential of overestimating the outcome measure; however, identifying severity from primary care data (EMIS) requires consistent recording of asthma medication within a structure such as the GINA criteria.^40^ This was introduced, within EMIS in 2020, after the study period and therefore could not be included in the analysis. In addition, the use of proxy data, specifically oral corticosteroid use, as a surrogate for severity can overestimate this.^41^

The outcome of a coded AE was assumed to be an independent event; however, repeated attendances, if documented as a new event, would overestimate the AE incidence rate. Conversely adults with severe asthma who bypass primary care to attend secondary care or adults with mild asthma who self-medicate will not be recorded in primary care data leading to an underestimation of incidence rate.

### Implications

The increase in AE incidence rate for the asthma population with comorbid mental health disorder diagnoses as shown in this study is higher than previously reported. Therefore, primary care service utilisation is proportionately greater for this population than those without comorbid mental health disorders. Consequently, initiatives to reduce AE incidence rate will have a greater impact if focused on this group.

## Conclusion

This study adds to the literature on asthma outcomes for patients with asthma and mental health disorders by confirming an association between the presence of mental health disorders and a relative increase in asthma exacerbation incidence rate compared with asthma patients without mental health disorders. Although it cannot prove causality, this study highlights the extra burden of AE on those with comorbid depression and anxiety.

## Supplementary material

10.1136/bmjresp-2025-003244 online supplemental file 1

## Data Availability

No data are available.
