# Supplementary material for: Association between mental health disorders and asthma exacerbations in adults: a retrospective cohort study in UK primary care
Source: BMJ Open Respir Res. 2025 Nov 17;12(1):e003244. doi: 10.1136/bmjresp-2025-003244 (PMC12625874; doi:10.1136/bmjresp-2025-003244)
Supplement: online supplemental file 1 [file bmjresp-12-1-s001.docx]

Appendix 1: SNOMED CT codes

| Asthma exacerbation codes | SNOMED_CT_CODE |
| --- | --- |
| Infrequent asthma exacerbations | 1821511000006100 |
| Frequent asthma exacerbations | 1821531000006100 |
| Acute non-infective exacerbation of asthma | 2010041000006100 |
| Extrinsic asthma with asthma attack | 708093000 |
| Asthma attack NOS | 708038006 |
| Acute exacerbation of asthma | 708038006 |
| Severe asthma attack | 708090002 |
| Life threatening acute exacerbation of non-allergic asthma | 1086711000000100 |
| Acute exacerbation of extrinsic asthma | 708093000 |
| Acute exacerbation of asthma | 708038006 |
| Acute severe exacerbation of asthma | 708090002 |
| Life threatening acute exacerbation of extrinsic asthma | 1086701000000100 |
| Acute exacerbation of immunoglobulin E-mediated allergic asthma | 708093000 |
| Occasional asthma exacerbations | 1821521000006100 |
| Severe asthma attack | 708038006 |
| Acute infective exacerbation of asthma | 2010031000006100 |
| Life threatening acute exacerbation of intrinsic asthma | 1086711000000100 |
| Life threatening acute exacerbation of allergic asthma | 1086701000000100 |
| Asthma attack | 708038006 |
| Intrinsic asthma with asthma attack | 708094006 |
| Exacerbation of asthma | 281239006 |
| Moderate acute exacerbation of asthma | 734905008 |
| Chronic obstructive asthma co-occurrent with acute exacerbation of asthma | 10692721000119100 |
| Acute severe exacerbation of allergic asthma | 708095007 |

| Depression codes | SNOMED_CT_CODE |
| --- | --- |
| Agitated depression | 83458005 |
| Senile dementia with depression | 191459006 |
| Arteriosclerotic dementia with depression | 191466007 |
| Single major depressive episode, severe, with psychosis | 191604000 |
| Recurrent major depressive episodes, mild | 191610000 |
| Recurrent major depressive episodes, moderate | 191611001 |
| Recurrent major depressive episodes, severe, with psychosis | 191613003 |
| Reactive depressive psychosis | 191676002 |
| Psychotic reactive depression | 191676002 |
| Postviral depression | 192079006 |
| Endogenous depression first episode | 231499006 |
| Masked depression | 231500002 |
| Seasonal affective disorder | 247803002 |
| Endogenous depression - recurrent | 274948002 |
| Depression management programme | 401174001 |
| On depression register | 413169006 |
| Patient given advice about management of depression | 415044007 |
| Depression interim review | 413973005 |
| Depression medication review | 413974004 |
| Depression annual review | 413972000 |
| [X]Mild depression | 310495003 |
| Referral for guided self-help for depression | 199111000000000 |
| Depression monitoring first letter | 717211000000000 |
| Depression monitoring third letter | 716961000000000 |
| On full dose long term treatment for depression | 361761000000000 |
| Post natal depression | 853871000000000 |
| Reactive (neurotic) depression | 87414006 |
| [RFC] Postnatal depression | 908731000000000 |
| Antenatal depression | 1771530000000000 |
| Unspecified dementia, other symptoms, predominantly depressive | 1972070000000000 |
| Organic depressive disorder | 1972110000000000 |
| Dementia in Alzheimer's dis, atypical or mixed type, other symptoms, predominantly depressive | 1972310000000000 |
| Dementia in Alzheimer's disease, unspecified, other symptoms, predominantly depressive | 1972450000000000 |
| Vascular dementia of acute onset, other symptoms, predominantly depressive | 1972540000000000 |
| Mixed cortical and subcortical vascular dementia, other symptoms, predominantly depressive | 1972910000000000 |
| Other vascular dementia, other symptoms, predominantly depressive | 1973380000000000 |
| Post-schizophrenic depression, continuous | 1975190000000000 |
| Post-schizophrenic depression, episodic with progressive deficit | 1975210000000000 |
| Post-schizophrenic depression, episodic remittent | 1975260000000000 |
| Depression codes | SNOMED_CT_CODE |
| Post-schizophrenic depression, course uncertain, period of observation too short | 1975320000000000 |
| Mild depressive episode, without somatic syndrome | 1975980000000000 |
| Moderate depressive episode, without somatic syndrome | 1976020000000000 |
| Recurrent depressive disorder, current episode mild, with somatic syndrome | 1976230000000000 |
| Recurrent depressive disorder, current episode moderate, with somatic syndrome | 1976270000000000 |
| Mixed anxiety and depressive reaction | 1976490000000000 |
| Post-schizophrenic depression, other | 1976920000000000 |
| Maternal postnatal depression | 1038260000000000 |
| Signposting to depression self-help group | 1057350000000000 |
| Senile dementia with depressive or paranoid features NOS | 191457008 |
| Recurrent major depressive episodes, unspecified | 268621008 |
| Brief depressive reaction NOS | 192046006 |
| Depressive disorder NEC | 35489007 |
| [X]Other recurrent depressive disorders | 191616006 |
| [X]Other depressive episodes | 35489007 |
| [X]Depressive episode, unspecified | 35489007 |
| [X]Recurrent depressive disorder | 191616006 |
| [X]Recurrent depressive disorder, unspecified | 191616006 |
| Prolonged depressive reaction | 192049004 |
| [X] Reactive depression NOS | 87414006 |
| [X]Depressive conduct disorder | 231542000 |
| [X]Depressive episode | 35489007 |
| [X]Depressive neurosis | 78667006 |
| [X]Dysthymia | 78667006 |
| [X]Mild anxiety depression | 231504006 |
| [X]Mixed anxiety and depressive disorder | 231504006 |
| [X]Monopolar depression NOS | 35489007 |
| [X]Neurotic depression | 78667006 |
| Depression monitoring telephone invite | 716421000000000 |
| [X]Post-schizophrenic depression | 231485007 |
| [X]Postnatal depression NOS | 58703003 |
| [X]Postpartum depression NOS | 58703003 |
| [X]Recurr severe episodes/psychogenic depressive psychosis | 191613003 |
| [X]Recurrent brief depressive episodes | 40568001 |
| [X]Recurrent depress disorder cur epi severe with psyc symp | 28475009 |
| [X]Recurrent episodes of depressive reaction | 191616006 |
| [X]Recurrent episodes of psychogenic depression | 191616006 |
| [X]Recurrent episodes of reactive depression | 191616006 |
| [X]Schizoaffective disorder, depressive type | 84760002 |
| [X]Schizoaffective psychosis, depressive type | 84760002 |
| Depression codes | SNOMED_CT_CODE |
| [X]Seasonal depressive disorder | 247803002 |
| [X]Single episode of masked depression NOS | 231500002 |
| [X]Single episode of reactive depression | 87414006 |
| Anxiety with depression | 231504006 |
| Brief depressive reaction | 192046006 |
| Depressive psychoses | 35489007 |
| [X]Major depression, severe with psychotic symptoms | 73867007 |
| [X]Major depression, severe without psychotic symptoms | 75084000 |
| [X]Antenatal depression | 790961000000000 |
| Recurrent major depressive episode NOS | 268621008 |
| [X]Recurrent depressive disorder, current episode mild | 310495003 |
| [X]Recurrent depressive disorder, current episode moderate | 310496002 |
| [D]Postoperative depression | 82218004 |
| Recurrent major depressive episodes, severe, no psychosis | 764611000000000 |
| [X]Persistent anxiety depression | 231504006 |
| [X]Prolonged single episode of reactive depression | 87414006 |
| [X]Single episode of psychogenic depression | 87414006 |
| [X]Single episode of reactive depressive psychosis | 191676002 |
| [X]Mild depressive episode | 310495003 |
| [X]Moderate depressive episode | 310496002 |
| Single major depressive episode | 36923009 |
| [X]Severe depressive episode without psychotic symptoms | 310497006 |
| [X]Single episode major depression w'out psychotic symptoms | 310497006 |
| [X]Recurrent severe episodes/reactive depressive psychosis | 1086470000000000 |
| Single major depressive episode, severe, without psychosis | 251000000000 |
| Mild depression | 310495003 |
| Major depressive disorder, single episode | 36923009 |
| History of depression | 161469008 |
| Single major depressive episode, moderate | 15639000 |
| Counselling for postnatal depression | 395072006 |
| [X]Recurrent severe episodes/reactive depressive psychosis | 191613003 |
| Psychotic depression | 73867007 |
| Moderate depression | 310496002 |
| Counselling for postnatal depression | 395072006 |
| Depressive conduct disorder | 231542000 |
| Postoperative depression | 82218004 |
| Depression management program | 401174001 |
| Recurrent reactive depressive episodes, severe, with psychosis | 1086470000000000 |
| Severe recurrent major depression with psychotic features | 28475009 |
| Schizophreniform psychosis, depressive type | 84760002 |
| Depressive illness | 35489007 |
| Recurrent brief depressive disorder | 40568001 |
| Post-schizophrenic depression | 231485007 |
| Depression codes | SNOMED_CT_CODE |
| History of depressive disorder | 161469008 |
| Depressive disorder NEC | 609311000000000 |
| Depression | 609311000000000 |
| Depression NOS | 609311000000000 |
| Moderate depression | 465441000000000 |
| Severe depression | 397701000000000 |
| Depression care management | 784051000000000 |
| [X]Mild depressive episode | 430421000000000 |
| Referral for guided self-help for depression declined | 933441000000000 |
| Dementia in Alzheimer's disease with early onset, other symptoms, predominantly depressive | 1972130000000000 |
| Multi-infarct dementia, other symptoms, predominantly depressive | 1972660000000000 |
| Recurrent depressive disorder, current episode moderate, without somatic syndrome | 1976250000000000 |
| Depression resolved | 196381000000000 |
| Atypical depressive disorder | 191659001 |
| Depression - enhanced service completed | 166481000000000 |
| [X]Depression NOS | 35489007 |
| [X]Depressive disorder NOS | 35489007 |
| [X]Recurr severe episodes/major depression+psychotic symptom | 28475009 |
| [X]SAD - Seasonal affective disorder | 247803002 |
| Neurotic depression reactive type | 87414006 |
| On full dose long term treatment depression - enh serv admin | 361761000000000 |
| [X]Severe depressive episode with psychotic symptoms | 191604000 |
| [X]Endogenous depression with psychotic symptoms | 73867007 |
| [X]Major depression, recurrent without psychotic symptoms | 268621008 |
| [X]Recurr depress disorder cur epi severe without psyc sympt | 310497006 |
| [X]Single episode of major depression and psychotic symptoms | 191604000 |
| [X]Single episode of psychogenic depressive psychosis | 191676002 |
| Severe depression | 310497006 |
| Depression care management | 784051000000000 |
| [X]Severe depressive episode without psychotic symptoms | 397701000000000 |
| QOF (Quality and Outcomes Framework) depression quality indicator-related care invitation | 1110910000000000 |
| Referral for depression self-help video | 923921000000000 |
| Dementia in Alzheimer's disease with late onset, other symptoms, predominantly depressive | 1972200000000000 |
| Subcortical vascular dementia, other symptoms, predominantly depressive | 1972770000000000 |
| Mild depressive episode, with somatic syndrome | 1975990000000000 |
| Moderate depressive episode, with somatic syndrome | 1976050000000000 |
| Recurrent depressive disorder, current episode mild, without somatic syndrome | 1976210000000000 |
| Depression confirmed | 1823880000000000 |
| Depression codes | SNOMED_CT_CODE |
| [RFC] Depression | 909681000000000 |
| Postnatal depression discussed | 939961000000000 |
| Depression monitoring administration | 713831000000000 |
| Depression monitoring second letter | 716681000000000 |
| Endogenous depression | 300706003 |
| Drug-induced depressive state | 191495003 |
| Recurrent depression | 191616006 |
| Postnatal depression | 58703003 |
| Depression monitoring verbal invite | 717261000000000 |
| [X]Recurrent severe episodes of psychotic depression | 191613003 |
| Agitated depression | 83458005 |
| Endogenous depression first episode | 231499006 |
| [X]Major depression, moderately severe | 832007 |
| [X]Major depression, mild | 87512008 |
| [X]Endogenous depression without psychotic symptoms | 300706003 |
| [X]Vital depression, recurrent without psychotic symptoms | 310497006 |
| Single major depressive episode, mild | 79298009 |
| Single major depressive episode, moderate | 15639000 |
| Single major depressive episode, unspecified | 36923009 |
| [X]Single episode vital depression w'out psychotic symptoms | 310497006 |
| Severe recurrent major depression without psychotic features | 36474008 |
| Drug-induced depression | 191495003 |
| Severe major depression, single episode | 251000000000 |
| Vascular dementia, unspecified, other symptoms, predominantly depressive | 1973550000000000 |
| Post-schizophrenic depression, episodic with stable deficit | 1975230000000000 |
| Other recurrent mood affective disorders, recurrent brief depressive disorder | 1976410000000000 |
| Moderate major depression, single episode | 15639000 |
| Puerperal depression | 58703003 |
| Postnatal depression counselling | 395072006 |
| Presenile dementia with depression | 191455000 |
| Senile dementia with depressive or paranoid features | 191457008 |
| Chronic depression | 192080009 |
| H/O: depression | 161469008 |
| Recurrent major depressive episode | 268621008 |
| Depression - enhanced services administration | 166291000000000 |
| Major depression, single episode | 36923009 |
| [X]Schizophreniform psychosis, depressive type | 84760002 |
| [X]Single episode of psychotic depression | 191604000 |
| [X]Atypical depression | 191659001 |
| [X]Single episode of depressive reaction | 87414006 |
| Single major depressive episode NOS | 36923009 |
| Depression codes | SNOMED_CT_CODE |
| [X]Single episode agitated depressn w'out psychotic symptoms | 310497006 |
| Postnatal depression counselling | 395072006 |
| Anxiety depression | 231504006 |
| Mild major depression, single episode | 79298009 |
| [X]Moderate depressive episode | 465441000000000 |
| Mild depression | 430421000000000 |

Anxiety codes

| Anxiety codes | SNOMED_CT_CODE |
| --- | --- |
| Dental phobia | 38617005 |
| Simple phobia | 54587008 |
| Acrophobia | 58963008 |
| H/O: anxiety state | 161470009 |
| Recurrent anxiety | 191709001 |
| Social phobia, fear of eating in public | 191724005 |
| Social phobia, fear of public speaking | 191725006 |
| Anxiety management training | 228560001 |
| Flying phobia | 247854002 |
| Generalised anxiety disorder | 21897009 |
| Cancer phobia | 34563004 |
| Panic disorder | 371631005 |
| Animal phobia | 54307006 |
| H/O: agoraphobia | 414371008 |
| Referral for guided self-help for anxiety | 199101000000102 |
| Anxious | 48694002 |
| Phonophobia | 851141000006108 |
| School phobia | 851351000006108 |
| [RFC] Anxiety management | 909691000006109 |
| Needle phobia | 54587008 |
| ** The treatment of anxiety disorders | 972931000006101 |
| Panic disorder without agoraphobia | 56576003 |
| Referral for guided self-help for anxiety declined | 933461000000100 |
| Referral for psychological management of anxiety | 1037451000000100 |
| Anxiety state unspecified | 198288003 |
| Anxiety state NOS | 198288003 |
| Agoraphobia without mention of panic attacks | 61569007 |
| [X]Phobic anxiety disorders | 386810004 |
| [X]Phobic anxiety disorder, unspecified | 386810004 |
| [X]Other anxiety disorders | 197480006 |
| [X]Separation anxiety disorder of childhood | 11806006 |
| [X]Agoraphobia | 70691001 |
| [X]Agoraphobia without history of panic disorder | 70691001 |
| [X]Animal phobias | 54307006 |
| [X]Anxiety hysteria | 197480006 |
| [X]Anxiety neurosis | 207363009 |
| [X]Childhood overanxious disorder | 13438001 |
| [X]Dream anxiety disorder | 419145002 |
| [X]Generalized anxiety disorder | 21897009 |
| [X]Mild anxiety depression | 231504006 |
| [X]Mixed anxiety and depressive disorder | 231504006 |
| [X]Nosophobia | 18193002 |
| [X]Organic anxiety disorder | 17496003 |
| Anxiety codes | SNOMED_CT_CODE |
| [X]Panic state | 371631005 |
| [X]Social anxiety disorder of childhood | 64165008 |
| [X]Social phobias | 25501002 |
| Anxiety with depression | 231504006 |
| Disturbance anxiety and fearfulness childhood/adolescent NOS | 192108001 |
| [X]Needle phobia | 231501003 |
| [X]Other phobic anxiety disorders | 386810004 |
| [X]Persistant anxiety depression | 231504006 |
| [X]Simple phobia | 386810004 |
| Anxiety states | 197480006 |
| Management of anxiety | 710060004 |
| Adjustment disorder with anxiety | 47372000 |
| AMT - Anxiety management training | 228560001 |
| History of agoraphobia | 414371008 |
| Phobia of going out | 70691001 |
| History of anxiety state | 161470009 |
| Adjustment disorder with anxious mood | 47372000 |
| Episodic paroxysmal anxiety disorder | 371631005 |
| [X]Phobia NOS | 386810004 |
| Social anxiety disorder | 25501002 |
| Feeling anxious | 48694002 |
| Anxious cognitions | 79015004 |
| Anxiety counseling | 313087008 |
| GAD - Generalised anxiety disorder | 21897009 |
| Anxiety | 48694002 |
| Isolated phobia | 54587008 |
| Counseling for anxiety | 313087008 |
| Generalized anxiety disorder | 21897009 |
| GAD - Generalized anxiety disorder | 21897009 |
| Agoraphobia | 191723004 |
| Needle phobia | 563201000000101 |
| Agoraphobia without mention of panic attacks | 191723004 |
| Referral for psychological management of anxiety declined | 1037471000000100 |
| Feeling anxious | 1818111000006100 |
| Agoraphobia | 191722009 |
| O/E - anxious | 162723006 |
| [X]Anxiety disorder, unspecified | 197480006 |
| [X]Acrophobia | 54587008 |
| [X]Anxiety NOS | 197480006 |
| [X]Phobic anxiety disorder of childhood | 192611004 |
| Disturbance of anxiety and fearfulness childhood/adolescent | 192108001 |
| Counselling for anxiety | 313087008 |
| Anxiety codes | SNOMED_CT_CODE |
| On examination - anxious | 162723006 |
| Phobia unspecified | 386808001 |
| [X]Panic disorder [episodic paroxysmal anxiety]  Breathlessness causing anxiety | 371631005  1861181000006100 |
| Phobic anxiety | 853241000006103 |
| Chronic anxiety | 191708009 |
| Social phobia, fear of public washing | 191726007 |
| Organic anxiety disorder | 17496003 |
| [X]Anthropophobia | 25501002 |
| [X]Anxious [avoidant] personality disorder | 231528008 |
| [X]Panic disorder with agoraphobia | 191722009 |
| Overanxious disorder of childhood | 13438001 |
| Zoophobia | 54307006 |
| [X]Other mixed anxiety disorders | 231504006 |
| Anxiety resolved | 1037391000000100 |
| Separation anxiety disorder | 11806006 |
| Agoraphobia with panic attacks | 191722009 |
| Claustrophobia | 19887002 |
| [X]Other specified anxiety disorders | 197480006 |
| Phobic anxiety | 386810004 |
| Childhood phobic anxiety disorder | 192611004 |
| [X]Anxiety state | 198288003 |
| [X]Claustrophobia | 54587008 |
| [X]Phobia NOS | 386810004 |
| [X]Specific (isolated) phobias | 54587008 |
| Anxiety counselling | 313087008 |
| Anxiety depression | 231504006 |
| Cyesiophobia | 191733007 |
| [X]Organic anxiety disorder | 17496003 |
| Other phobias | 563201000000101 |
| Anxiety state | 198288003 |

Bipolar codes

| Bipolar codes | SNOMED_CT_CODE |
| --- | --- |
| Single manic episode, mild | 191583000 |
| Single manic episode, moderate | 191584006 |
| Single manic episode, severe, with psychosis | 191586008 |
| Single manic episode in full remission | 191588009 |
| Recurrent manic episodes | 191590005 |
| Recurrent manic episodes, mild | 191592002 |
| Recurrent manic episodes, moderate | 191593007 |
| Recurrent manic episodes, severe, with psychosis | 191595000 |
| Manic-depressive - now manic | 191618007 |
| Bipolar affective disorder, currently manic, mild | 191620005 |
| Manic-depressive - now depressed | 191627008 |
| Bipolar affective disorder, currently depressed, mild | 191629006 |
| Mixed bipolar affective disorder | 191636007 |
| Mixed bipolar affective disorder, in full remission | 191643001 |
| Manic disorder, single episode | 268619003 |
| H/O: manic depressive disorder | 400998002 |
| Bipolar affective disorder resolved | 285491000000000 |
| Organic bipolar affective disorder | 1972100000000000 |
| Bipolar affect disorder cur epi mild or moderate depressn, with somatic syndrome | 1975940000000000 |
| Single manic episode, unspecified | 268619003 |
| Bipolar affective disorder, currently manic, unspecified | 191618007 |
| Bipolar affective disorder, currently manic, NOS | 191618007 |
| Bipolar affective disorder, currently depressed, unspecified | 191627008 |
| Bipolar affective disorder, currently depressed, NOS | 191627008 |
| Unspecified bipolar affective disorder | 13746004 |
| Unspecified bipolar affective disorder, unspecified | 13746004 |
| Unspecified bipolar affective disorder, mild | 13313007 |
| Unspecified bipolar affective disorder, NOS | 13746004 |
| Other and unspecified manic-depressive psychoses | 13746004 |
| [X]Bipolar affective disorder, currently in remission | 85248005 |
| [X]Mania with psychotic symptoms | 231494001 |
| [X]Manic episode, unspecified | 268619003 |
| [X]Other bipolar affective disorders | 13746004 |
| [V]Personal history of manic-depressive psychosis | 429124005 |
| [V]Personal history of manic-depressive psychosis | 429124005 |
| [X]Bipolar affective disorder | 13746004 |
| [X]Bipolar affect disorder cur epi manic wout psychotic symp | 191618007 |
| [X]Bipolar affective disorder, current episode hypomanic | 31446002 |
| [X]Bipolar affective disorder, current episode mixed | 192362008 |
| [X]Bipolar disorder, single manic episode | 268619003 |
| [X]Bipolar II disorder | 83225003 |
| [X]Cyclothymia | 76105009 |

| Bipolar codes | SNOMED_CT_CODE |
| --- | --- |
| [X]Manic-depressive reaction  [X]Mania NOS | 13746004  268619003 |
| [X]Mania with mood-incongruent psychotic symptoms | 231494001 |
| [X]Manic episode | 268619003 |
| [X]Manic-depressive illness | 13746004 |
| [X]Manic-depressive psychosis | 13746004 |
| Bipolar affect disord, currently manic,severe with psychosis | 191623007 |
| Bipolar affect disord, now depressed, severe, no psychosis | 61403008 |
| Bipolar affective disorder, currently depressed | 191627008 |
| Bipolar affective disorder, currently manic | 191618007 |
| Bipolar affective disorder, currently manic, full remission | 191625000 |
| Bipolar psychoses | 13746004 |
| [X]Bipolar affective disorder type I | 371596008 |
| [X]Bipolar II disorder | 83225003 |
| Single manic episode, severe without mention of psychosis | 764641000000000 |
| Mixed bipolar affective disorder, NOS | 191636007 |
| Unspecified bipolar affective disorder, moderate | 79584002 |
| Unspecified bipolar affective disorder, in full remission | 41836007 |
| Other mixed manic-depressive psychoses | 16506000 |
| Unspecified bipolar affect disord, partial/unspec remission | 5703000 |
| Recurrent manic episodes, partial or unspecified remission | 764671000000000 |
| Recurrent manic episodes, severe without mention psychosis | 764621000000000 |
| [X]Bipolar affect disorder cur epi manic with psychotic symp | 191623007 |
| [X]Bipolar affect disorder cur epi mild or moderate depressn | 191630001 |
| [X]Recurrent manic episodes | 191590005 |
| Bipolar affect disord, now depressed, part/unspec remission | 49512000 |
| Mixed bipolar affective disorder, partial/unspec remission | 760721000000000 |
| Mixed bipolar affective disorder, severe, without psychosis | 764591000000000 |
| Manic-depressive psychoses | 13746004 |
| [X]Bipolar affect dis cur epi severe depres with psyc symp | 765176007 |
| Bipolar affect disord, now depressed, severe with psychosis | 765176007 |
| [X]Manic-depress psychosis,depressed type+psychotic symptoms | 765176007 |
| Bipolar disorder, in remission | 85248005 |
| Severe bipolar I disorder, most recent episode manic, without psychotic features | 162004 |
| Bipolar I disorder, most recent episode depressed, in partial remission | 49512000 |
| Severe bipolar I disorder, most recent episode depressed without psychotic features | 61403008 |
| Bipolar I disorder, most recent episode mixed | 16506000 |
| Mixed bipolar affective disorder, in partial remission | 760721000000000 |
| Bipolar affective disorder, current episode mixed | 192362008 |
| History of manic depressive disorder | 400998002 |
| Bipolar 2 disorder | 83225003 |
| Bipolar disorder, partial remission | 5703000 |
|  |  |
| Bipolar codes | SNOMED_CT_CODE |
| Bipolar affect disorder cur epi mild or moderate depressn, without somatic syndrome | 1975930000000000 |
| Mixed bipolar affective disorder, severe, with psychosis  Recurrent manic episode NOS | 191641004  191590005 |
| Unspecified manic-depressive psychoses | 13746004 |
| [X]Mania without psychotic symptoms | 231494001 |
| Bipolar affect disord, currently manic, severe, no psychosis | 162004 |
| Bipolar affective disorder, now depressed, in full remission | 191634005 |
| Psychosis, schizophrenia + bipolar affective disord resolved | 200951000000000 |
| [X]Other manic episodes | 231494001 |
| MDI - Manic-depressive illness | 13746004 |
| Bipolar 1 disorder | 371596008 |
| Bipolar I disorder, most recent episode manic, in partial remission | 63249007 |
| Manic disorder, single episode NOS | 268619003 |
| Recurrent manic episodes, in full remission | 191597008 |
| Bipolar affective disorder, currently depressed, moderate | 191630001 |
| [X]Bipol aff disord, curr epis sev depress, no psychot symp | 61403008 |
| Unspecified bipolar affective disorder,severe with psychosis | 4441000 |
| [X]Mania with mood-congruent psychotic symptoms | 231494001 |
| Bipolar affect disord,currently manic, part/unspec remission | 63249007 |
| [X]Bipolar affective disorder type II | 83225003 |
| Single manic episode in partial or unspecified remission | 764731000000000 |
| Unspecified bipolar affective disorder, severe, no psychosis | 53049002 |
| Bipolar disorder, full remission | 41836007 |
| Recurrent manic episodes, unspecified | 191590005 |
| Other and unspecified manic-depressive psychoses NOS | 13746004 |
| [X]Bipolar affective disorder, unspecified | 13746004 |
| Bipolar affective disorder, currently manic, moderate | 191621009 |
| Mixed bipolar affective disorder, mild | 191638008 |
| Mixed bipolar affective disorder, moderate | 191639000 |
| [X]Manic-depress psychosis,depressd,no psychotic symptoms | 36474008 |
| Mixed bipolar affective disorder, unspecified | 191636007 |
| Bipolar I disorder, most recent episode hypomanic | 31446002 |

Schizophrenia codes

| Schizophrenia codes | SNOMED_CT_CODE |
| --- | --- |
| Chronic paranoid schizophrenia | 31658008 |
| Subchronic catatonic schizophrenia | 42868002 |
| Paranoid schizophrenia in remission | 63181006 |
| Paranoid schizophrenia | 64905009 |
| Chronic catatonic schizophrenia | 68995007 |
| Subchronic paranoid schizophrenia | 79866005 |
| FH: Schizophrenia | 160328009 |
| Schizophrenia association member | 161103001 |
| Schizophrenic disorders | 191526005 |
| Schizophrenia simplex | 191527001 |
| Simple schizophrenia | 191527001 |
| Catatonic schizophrenia | 191542003 |
| Acute exacerbation of subchronic catatonic schizophrenia | 191547009 |
| Acute exacerbation of chronic catatonic schizophrenia | 191548004 |
| Acute exacerbation of subchronic paranoid schizophrenia | 191554003 |
| Acute exacerbation of chronic paranoid schizophrenia | 191555002 |
| Latent schizophrenia | 191559008 |
| Chronic latent schizophrenia | 191562006 |
| Acute exacerbation of chronic latent schizophrenia | 191564007 |
| Latent schizophrenia in remission | 191565008 |
| Cyclic schizophrenia | 191567000 |
| Cenesthopathic schizophrenia | 191577003 |
| Atypical schizophrenia | 111484002 |
| Schizophrenic mother in law | 853501000000000 |
| Schizophrenic psychoses | 191526005 |
| Mental & behav dis due to vol solvents: psychotic disordr, schizophrenia-like | 1973050000000000 |
| Catatonic schizophrenia, continuous | 1974750000000000 |
| Ment/behav dis mlti drug use/oth psyc sbs: psychotc dis, schizophrenia-like | 1973830000000000 |
| Paranoid schizophrenia, continuous | 1974350000000000 |
| Paranoid schizophrenia, episodic with progressive deficit | 1974390000000000 |
| Paranoid schizophrenia, complete remission | 1974510000000000 |
| Paranoid schizophrenia, course uncertain, period of observation too short | 1974540000000000 |
| Hebephrenic schizophrenia, continuous | 1974600000000000 |
| Hebephrenic schizophrenia, episodic with progressive deficit | 1974610000000000 |
| Hebephrenic schizophrenia, episodic remittent | 1974640000000000 |
| Mental & behav dis due to use tobacco: psychotic disorder, schizophrenia-like | 1974650000000000 |
| Hebephrenic schizophrenia, complete remission | 1974690000000000 |
| Hebephrenic schizophrenia, course uncertain, period of observation too short | 1974720000000000 |
|  |  |
| Schizophrenia codes | SNOMED_CT_CODE |
| Catatonic schizophrenia, episodic with stable deficit  Catatonic schizophrenia, episodic remittent | 1974800000000000  1974830000000000 |
| Catatonic schizophrenia, incomplete remission | 1974860000000000 |
| Catatonic schizophrenia, course uncertain, period of observation too short | 1974920000000000 |
| Undifferentiated schizophrenia, continuous | 1974950000000000 |
| Undifferentiated schizophrenia, episodic with progressive deficit | 1974970000000000 |
| Undifferentiated schizophrenia, episodic with stable deficit | 1975000000000000 |
| Undifferentiated schizophrenia, episodic remittent | 1975020000000000 |
| Post-schizophrenic depression, continuous | 1975190000000000 |
| Post-schizophrenic depression, episodic with progressive deficit | 1975210000000000 |
| Post-schizophrenic depression, episodic remittent | 1975260000000000 |
| Post-schizophrenic depression, complete remission | 1975300000000000 |
| Post-schizophrenic depression, course uncertain, period of observation too short | 1975320000000000 |
| Residual schizophrenia, continuous | 1975350000000000 |
| Residual schizophrenia, episodic with progressive deficit | 1975380000000000 |
| Residual schizophrenia, episodic with stable deficit | 1975400000000000 |
| Residual schizophrenia, episodic remittent | 1975430000000000 |
| Residual schizophrenia, incomplete remission | 1975440000000000 |
| Simple schizophrenia, continuous | 1975490000000000 |
| Simple schizophrenia, episodic with stable deficit | 1975520000000000 |
| Mental and behav dis due to other stimulants inc caffeine: psychotic dis, schizophrenia-like | 1975560000000000 |
| Simple schizophrenia, incomplete remission | 1975570000000000 |
| Simple schizophrenia, complete remission | 1975600000000000 |
| Acute schizophrenia-like psychotic disorder, without associated acute stress | 1975710000000000 |
| Acute schizophrenia-like psychotic disorder, with associated acute stress | 1975730000000000 |
| Other schizophrenia, continuous | 1975750000000000 |
| Other schizophrenia, episodic with stable deficit | 1975800000000000 |
| Other schizophrenia, complete remission | 1975880000000000 |
| Other schizophrenia, course uncertain, period of observation too short | 1975910000000000 |
| Schizophrenia, unspecified, episodic with progressive deficit | 1976060000000000 |
| Schizophrenia, unspecified, episodic with stable deficit | 1976100000000000 |
| Schizophrenia, unspecified, complete remission | 1976150000000000 |
| Schizophrenia, unspecified, course uncertain, period of observation too short | 1976170000000000 |
| Paranoid schizophrenia, other | 1976880000000000 |
| Post-schizophrenic depression, other | 1976920000000000 |
| Residual schizophrenia, other | 1976930000000000 |
| Simple schizophrenia, other | 1976940000000000 |
| Schizophrenia, unspecified, other | 1976960000000000 |
| Schizophrenia codes | SNOMED_CT_CODE |
| Subchronic hebephrenic schizophrenia | 27387000 |
| Chronic hebephrenic schizophrenia | 12939007 |
| Hebephrenic schizophrenia in remission | 31373002 |
| Unspecified schizophrenia | 58214004 |
| Unspecified hebephrenic schizophrenia  Catatonic schizophrenia NOS | 35252006  191542003 |
| Other schizophrenia NOS | 58214004 |
| Schizophrenia NOS | 58214004 |
| [X]Undifferentiated schizophrenia | 111484002 |
| [X]Schizophrenia, unspecified | 58214004 |
| Schizophrenic child | 444180005 |
| Unspecified schizo-affective schizophrenia | 191567000 |
| Schizo-affective schizophrenia | 191567000 |
| Schizo-affective schizophrenia in remission | 191574005 |
| Schizo-affective schizophrenia NOS | 191567000 |
| Restzustand - schizophrenia | 26025008 |
| [X]Acute schizophrenia-like psychotic disorder | 278853003 |
| [X]Atypical schizophrenia | 111484002 |
| [X]Bouffee delirante with symptoms of schizophrenia | 712850003 |
| [X]Cenesthopathic schizophrenia | 58214004 |
| [X]Disorganised schizophrenia | 35252006 |
| [X]Hebephrenic schizophrenia | 35252006 |
| [X]Mixed schizophrenic and affective psychosis | 270901009 |
| [X]Paranoid schizophrenia | 64905009 |
| [X]Post-schizophrenic depression | 231485007 |
| [X]Pseudopsychopathic schizophrenia | 31027006 |
| [X]Residual schizophrenia | 26025008 |
| [X]Schizophrenia | 58214004 |
| [X]Schizophrenia-like psychosis in epilepsy | 5510009 |
| [X]Schizophrenic catalepsy | 191542003 |
| [X]Schizophrenic catatonia | 191542003 |
| [X]Schizophrenic reaction | 278853003 |
| [X]Simple schizophrenia | 191527001 |
| Acute exacerbation of chronic schizo-affective schizophrenia | 191572009 |
| Chronic schizo-affective schizophrenia | 191570001 |
| Hebephrenic schizophrenia | 35252006 |
| Unspecified latent schizophrenia | 191559008 |
| [X]Borderline schizophrenia | 274952002 |
| [X]Chronic undifferentiated schizophrenia | 111484002 |
| [X]Latent schizophrenia | 191559008 |
| [X]Prepsychotic schizophrenia | 247804008 |
| [V]Personal history of schizophrenia | 161464003 |
| Acute exacerbation of chronic schizoaffective schizophrenia | 191572009 |
| Schizoaffective schizophrenia, in remission | 191574005 |
| Schizophrenia assoc'n member | 161103001 |
| Schizophrenia codes | SNOMED_CT_CODE |
| Chronic catatonic schizophrenia with acute exacerbation | 191548004 |
| Schizophrenia, catatonic | 191542003 |
| Acute exacerbation of subchronic schizoaffective schizophrenia | 191571002 |
| Subchronic paranoid schizophrenia with acute exacerbation | 191554003 |
| Paranoid schizophrenia, in remission  Disorganized schizophrenia, in remission | 63181006  31373002 |
| Paraphrenic schizophrenia | 64905009 |
| Disorganized schizophrenia in remission | 31373002 |
| Mixed schizophrenic and affective psychosis | 270901009 |
| Schizophrenic prodrome | 247804008 |
| Acute polymorphic psychotic disorder with symptoms of schizophrenia | 712850003 |
| Subchronic disorganized schizophrenia with acute exacerbations | 14291003 |
| Schizophrenia, catatonic, in remission | 111483008 |
| History of schizophrenia | 161468000 |
| Subchronic disorganised schizophrenia | 27387000 |
| Post-schizophrenic depression | 231485007 |
| Schizophrenia, in remission | 4926007 |
| Subchronic disorganized schizophrenia | 27387000 |
| Subchronic disorganised schizophrenia with acute exacerbations | 14291003 |
| Chronic disorganised schizophrenia | 12939007 |
| Catatonic schizophrenia, complete remission | 1974890000000000 |
| Post-schizophrenic depression, incomplete remission | 1975280000000000 |
| Residual schizophrenia, course uncertain, period of observation too short | 1975480000000000 |
| Simple schizophrenia, episodic remittent | 1975550000000000 |
| Schizophrenia, unspecified, continuous | 1976000000000000 |
| Schizophrenia, unspecified, episodic remittent | 1976120000000000 |
| Catatonic schizophrenia, other | 1976900000000000 |
| Chronic schizophrenic | 83746006 |
| Residual schizophrenia | 26025008 |
| Catatonic schizophrenia in remission | 111483008 |
| Unspecified paranoid schizophrenia | 64905009 |
| [X]Cyclic schizophrenia | 270901009 |
| Acute exacerbation subchronic schizo-affective schizophrenia | 191571002 |
| Psychosis, schizophrenia + bipolar affective disord resolved | 200951000000000 |
| [X]Cycloid psychosis with symptoms of schizophrenia | 307417003 |
| Acute exacerbation of chronic disorganized schizophrenia | 191539009 |
| Disorganized schizophrenia | 35252006 |
| [X]Schizophrenia, schizotypal and delusional disorders | 417601000000000 |
| Unspecified catatonic schizophrenia | 191542003 |
| Paranoid schizophrenia NOS | 64905009 |
| Mental & behav dis due to use opioids: psychotic disorder, schizophrenia-like | 1971470000000000 |
|  |  |
| Schizophrenia codes | SNOMED_CT_CODE |
| Mental & behav dis due to hallucinogens: psychotic disord, schizophrenia-like | 1972010000000000 |
| Mental & behav dis due to cannabinoids: psychotic disordr, schizophrenia-like | 1972260000000000 |
| Mental & behav dis due to seds/hypntcs: psychotic disordr, schizophrenia-like | 1972860000000000 |
| Paranoid schizophrenia, episodic with stable deficit | 1974430000000000 |
| Hebephrenic schizophrenia, episodic with stable deficit | 1974620000000000 |
| Catatonic schizophrenia, episodic with progressive deficit  Undifferentiated schizophrenia, incomplete remission | 1974780000000000  1975050000000000 |
| Simple schizophrenia, course uncertain, period of observation too short | 1975640000000000 |
| Other schizophrenia, episodic remittent | 1975830000000000 |
| Other schizophrenia, incomplete remission | 1975840000000000 |
| Undifferentiated schizophrenia, other | 1976910000000000 |
| Schizophrenic psychoses NOS | 191526005 |
| Acute exacerbation of chronic schizophrenia | 191531007 |
| Subchronic latent schizophrenia | 191561004 |
| Acute exacerbation of subchronic latent schizophrenia | 191563001 |
| Coenesthopathic schizophrenia | 191577003 |
| [X]Schizophrenia, schizotypal and delusional disorders | 417601000000000 |
| H/O: schizophrenia | 161468000 |
| Childhood schizophrenia NOS | 191687005 |
| Subchronic schizo-affective schizophrenia | 191569002 |
| [X]Other schizophrenia | 58214004 |
| [X]Paraphrenic schizophrenia | 64905009 |
| [X]Restzustand schizophrenic | 26025008 |
| [X]Latent schizophrenic reaction | 191559008 |
| [X]Prodromal schizophrenia | 247804008 |
| Acute exacerbation of subchronic schizophrenia | 111482003 |
| Disorganised schizophrenia in remission | 31373002 |
| Latent schizophrenia, in remission | 191565008 |
| Schizophrenia, schizoaffective, in remission | 191574005 |
| Subchronic catatonic schizophrenia with acute exacerbation | 191547009 |
| Chronic paranoid schizophrenia with acute exacerbation | 191555002 |
| Chronic schizophrenia | 83746006 |
| Simple schizophrenia NOS | 191527001 |
| Hebephrenic schizophrenia NOS | 35252006 |
| Paranoid schizophrenia, episodic remittent | 1974450000000000 |
| Paranoid schizophrenia, incomplete remission | 1974480000000000 |
| Hebephrenic schizophrenia, incomplete remission | 1974670000000000 |
| Undifferentiated schizophrenia, complete remission | 1975080000000000 |
| Mental & behav dis due to use alcohol: psychotic disorder, schizophrenia-like | 1975140000000000 |
|  |  |
| Schizophrenia codes | SNOMED_CT_CODE |
| Undifferentiated schizophrenia, course uncertain, period of observation too short | 1975160000000000 |
| Post-schizophrenic depression, episodic with stable deficit | 1975230000000000 |
| Residual schizophrenia, complete remission | 1975470000000000 |
| Simple schizophrenia, episodic with progressive deficit | 1975500000000000 |
| Other schizophrenia, episodic with progressive deficit | 1975770000000000 |
| Schizophrenia, unspecified, incomplete remission | 1976140000000000 |
| Hebephrenic schizophrenia, other | 1976890000000000 |
| Other schizophrenia, other | 1976950000000000 |
| Acute schizophrenic episode | 268617001 |
| Acute exacerbation of chronic hebephrenic schizophrenia  [X]Schizophrenic flexibilatis cerea | 191539009  191542003 |
| Schizophrenia in remission | 4926007 |
| Subchronic schizophrenia | 16990005 |
| Other schizophrenia | 58214004 |
| Schizophrenic flexibilatis cerea | 191542003 |
| [X]Catatonic schizophrenia | 191542003 |
| [X]Organic delusional [schizophrenia-like] disorder | 5510009 |
| [X]Pseudoneurotic schizophrenia | 31027006 |
| Acute exacerbation of subchronic hebephrenic schizophrenia | 14291003 |
| Latent schizophrenia NOS | 191559008 |
| Acute exacerbation of chronic disorganised schizophrenia | 191539009 |
| Subchronic schizophrenia with acute exacerbations | 111482003 |
| Subchronic schizoaffective schizophrenia | 191569002 |
| Disorganised schizophrenia | 35252006 |
| Chronic disorganized schizophrenia | 12939007 |
